# Rotavirus Infection of Cells in Culture Induces Activation of RhoA and Changes in the Actin and Tubulin Cytoskeleton

**DOI:** 10.1371/journal.pone.0047612

**Published:** 2012-10-17

**Authors:** Jose Luis Zambrano, Orlando Sorondo, Ana Alcala, Esmeralda Vizzi, Yuleima Diaz, Marie Christine Ruiz, Fabian Michelangeli, Ferdinando Liprandi, Juan E. Ludert

**Affiliations:** 1 Instituto Venezolano de Investigaciones Científicas (IVIC), CMBC. Caracas, Venezuela; 2 Escuela de Biología, Universidad Central de Venezuela (UCV), Caracas, Venezuela; 3 University of Bergen Thormøhlensgate 55, Bergen, Norway; 4 Instituto Venezolano de Investigaciones Científicas (IVIC), CBB. Caracas, Venezuela; 5 Centro de Investigación y Estudios Avanzados del Instituto Politécnico Nacional (CINVESTAV-IPN), Ciudad de México, México; Instituto Gulbenkian de Ciência, Portugal

## Abstract

Rotavirus infection induces an increase in [Ca^2+^]_cyto_, which in turn may affect the distribution of the cytoskeleton proteins in the infected cell. Changes in microfilaments, including the formation of stress fibers, were observed starting at 0.5 h.p.i. using fluorescent phalloidin. Western blot analysis indicated that RhoA is activated between 0.5 and 1 h.p.i. Neither the phosphorylation of RhoA nor the formation of stress fibers were observed in cells infected with virions pre-treated with an anti-VP5* non-neutralizing mAb, suggesting that RhoA activation is stimulated by the interaction of the virus with integrins forming the cell receptor complex. In addition, the structure of the tubulin cytoskeleton was also studied. Alterations of the microtubules were evident starting at 3 h.p.i. and by 7 h.p.i. when microtubules were markedly displaced toward the periphery of the cell cytoplasm. Loading of rotavirus-infected cells with either a Ca^2+^ chelator (BAPTA) or transfection with siRNAs to silence NSP4, reversed the changes observed in both the microfilaments and microtubules distribution, but not the appearance of stress fibers. These results indicate that alterations in the distribution of actin microfilaments are initiated early during infection by the activation of RhoA, and that latter changes in the Ca^2+^ homeostasis promoted by NSP4 during infection may be responsible for other alterations in the actin and tubulin cytoskeleton.

## Introduction

Rotaviruses are icosahedral viruses, with 3 concentric protein layers containing the viral genome composed of 11 segments of dsRNA, grouped within the genus *Rotavirus* of the *Reoviridae* family [1]. Each genomic segment encodes for a single protein, with the exception of the smallest genomic segment, which encodes for 2 proteins (NSP5 and NSP6), for a total of 6 structural or viral proteins (VP1 to VP7) and 6 non-structural (NSP1 to NSP6) proteins [2]. The virion outer layer is composed of proteins VP7 and VP4, the intermediate layer of protein by VP6, and VP2 being the predominant inner core protein. Proteins VP1 and VP3 are part of the replication complex located within the inner core [2]. Non-structural proteins are synthesized in the infected cells and carry on functions during virus replication cycle and morphogenesis [1]. They also participate in the modulation of the innate immune response and pathogenesis [3].

The mature enterocyte is the main cell target for rotavirus replication in the host and gastroenteritis is the principal clinical outcome of rotavirus infection. The induction of diarrhea associated with rotavirus infections is thought to be multifactorial and to involve both malabsortive and secretory components [1], [3].

Direct cell damage caused by viral replication may be at the base of the malabsortive component, while the action of the viral enterotoxin NSP4 and stimulation of the enteric nervous system may be the main factors responsible for the secretory component of the diarrhea [4]. The NSP4 protein is a 28 kDa glycosylated integral endoplasmic reticulum (ER) membrane protein. NSP4 has the ability to interact with lipid membranes and to oligomerize to form tetramers [5]. At least 3 intracellular pools of NSP4 have been identified that localized to distinct sites within the cell, each with distinct functions [6]. During morphogenesis, ER resident NSP4 acts as a receptor for the nascent double layer particle in the viroplasm, and participates in the budding of the particles into the lumen of the ER. In infected cells where the expression of NSP4 is silenced, no binding of the viroplasm to the ER membrane is observed [7]. Moreover, NSP4 also affects intracellular membrane trafficking, mRNA synthesis and the expression of other viral proteins [8]–[11].

NSP4 or derived peptides are also secreted from infected cells via a non-classic secretory pathway [12]–[15]. Secreted NSP4 is capable of binding to neighboring, uninfected cells, using α1β1 and α2β1 integrins as receptors, and induces cell signaling pathways and intracellular Ca^2+^ mobilization [16]. Thus, it has become clear that NSP4 is a multifunctional protein with plays important roles both in virus morphogenesis and pathogenesis [11], [17].

The pathogenic potential of NSP4 is mainly associated with its ability to disrupt Ca^2+^ homeostasis both in infected and uninfected cells. However, important differences in the mechanism of Ca2^+^ mobilization have been observed when NSP4 is expressed endogenously, as during infection, or when it is added exogenously, as when released from infected cells. In rotavirus infected cells, NSP4 expression induces an increase in plasma membrane permeability to Ca^2+^ by an unknown phospholipase C (PLC) independent mechanism, which eventually leads to an elevation of cytosolic Ca^2+^ concentration ([Ca^2+^]_cyto_) [11], [13], [18], [19]. In parallel, the expression of NSP4 also provokes an increase of the total Ca^2+^ contained in the ER likely due to the secondary activation of SERCA pumps and a reduction of mobilizable ER pools, compatible with an increase of Ca^2+^ buffering capacity in this compartment [11], [19]–[21]. On the other hand, the binding of extracellular NSP4 to HT29 uninfected cells induces an increase of [Ca^2+^]_cyto_ linked to the release of Ca^2+^ from intracellular stores via a PLC and inositol tri-phosphate (IP3) dependent pathway [22], [23].

Disruption of both the actin and tubulin cytoskeleton has been observed in rotavirus infected-cells. Jourdan et al. (1998) reported that rotavirus infection induces microvillar F-actin disassembly in human polarized intestinal Caco-2 cells [24]. Alterations in F-actin in Caco-2 infected cells, and also in non-infected cells exposed to conditioned media from infected cells, were shown to be associated with changes in the intracellular Ca^2+^ concentration [25]. In agreement, Berkova et al. (2007) found that the transient expression of NSP4 resulted in an augmented F-actin content that was prevented if intracellular Ca^2+^ were normalized by incubation with low Ca^2+^ medium [26]. In addition, changes in the microtubule cytoskeleton of rotavirus infected Caco-2 cells were also observed as function of cell differentiation, and those changes also were found to be associated with alterations in cell Ca^2+^ homeostasis [25]. At variance, no changes in either actin or tubulin were observed in rotavirus infected neurons or CV-1 cells [27], [28]. Thus, changes in the cytoskeleton of rotavirus-infected cells may respond to multiple mechanisms and depend on cell type.

Despite considerable advancement, the mechanism of rotavirus diarrhea induction is not completely understood. In this work, using siRNAs to silence NSP4 expression evidence is presented which indicates that disruption of actin and tubulin cytoskeleton in rotavirus infected MA104 cells is directly related to changes in the intracellular Ca^2+^ concentration mediated by the expression of NSP4. Moreover, we present evidence that rotavirus infection induces the activation of the small GTPase RhoA and the formation of stress fibers, which are independent of Ca^2+^ changes. The disruption of the cell cytoskeleton not only may contribute to cell lysis but also to the disruption of tight junctions between infected enterocytes in the host with consequent paracellular permeability changes, thus adding to the massive induction of diarrhea observed during rotavirus infection.

## Materials and Methods

### Cell Culture and Virus Infection

The epithelial cell line MA104 from *Cercopithecus aethiops* kidney (American Type Culture Collection, ATCC; Manassas, Virginia) was growth in Eagle Minimal Essential Medium with Earlés salts, L-glutamine and sodium bicarbonate (MEM) (Gibco BRL; Invitrogen, Carlsbad, CA) supplemented with 10% fetal bovine serum (FBS) (Gibco) at 37°C in a 5% CO_2_ atmosphere. Cells were re-seeded and multiplied, every 3–4 days after detachment with 1% trypsin-EDTA solution (Sigma-Aldrich; ST Louis, MO).

Rotavirus DxRRV strain used throughout this work was kindly provided by H.B. Greenberg (Stanford University, Palo Alto, CA). The reassortant DxRRV contains gene 9 (encoding for rotavirus VP7 outer capsid structural protein) from parental human rotavirus D strain, on a genomic background of parental rotavirus RRV simian strain [7], [29]. The siRNA used in this work are designed to DxRRV genes (see below).

Infective titers of rotavirus DxRRV strain were measured by a Focus Forming Units (FFU/mL) assay using indirect immunocytochemistry staining. Briefly, DxRRV infectivity was activated with 10 µg/mL porcine trypsin (Sigma) for 1 h. Ten-fold dilutions of the virus were seeded on confluent monolayers of MA104 cells grown in 96 well-plates. After 16–18 h.p.i., cells were fixed with ice-cold methanol, washed with PBS and a monoclonal antibody (4B2D2) against antigen of rotavirus VP6 inner capsid structural protein was added as primary antibody [30]. The FFU were developed by 3,3′-diaminobenzidine tetrahydrochloride-peroxide (DAB) (Sigma) solution after incubation with an anti-mouse HRP-conjugated antibody (Sigma). Mock-infections were carried out in MA104 cells, following all the methods described but using PBS as inoculum.

### Immunofluorescence

MA104 cells were grown on cover glass circles (Thomas Scientific; Swedesboro, NJ) placed inside 12 well-plates. Cells were infected with rotavirus DxRRV with a m.o.i of 10 and at different times after infection (0.5, 1, 3, 5, 7 h.p.i.), cells were fixed with 4% paraformaldehyde (Sigma) in PBS for 7 min at room temperature (RT). Autofluorescence due to free aldehyde groups from paraformaldehyde treatment, were blocked with 50 mM Ammonium Chloride (Sigma) in PBS for 10 min at RT. Cells were permeabilized with 0.1% Triton X-100 (Sigma) in PBS for 7 min at RT and blocked with 1–4% bovine serum albumin (BSA) (Sigma) in PBS for 1 h at RT. Between each step described above, cells were washed twice with PBS for 5 min at RT. Viral proteins were stained with 4B2D2 and B42 as a primary mAb (VP6 and NSP4 proteins respectively, B42 antibody was kindly provided by H.B. Greenberg), followed by an anti-mouse rhodamine or anti-mouse Alexa 488 conjugated antibodies (Pierce, Thermo Fisher Scientific Inc; Rockford, IL). Actin filaments were labeled with Alexa Fluor 488 or Alexa Fluor 568 Phalloidin (Molecular Probe, Invitrogen) for 20 min at 37°C. Nuclei were stained for 3–5 min at room temperature with 4′6-Diamidine-2-phenylindole dilactate (DAPI) (Molecular Probes, Invitrogen). Microtubules were detected with an anti-α-tubulin mAb (Calbiochem Merck KGaA; Darmstadt, Germany) as primary antibody followed by an anti-mouse FITC conjugated antibody (Pierce, Thermo Fisher Scientific Inc; Rockford, IL). Finally, cells were washed three times with PBS for 5 min at RT and mounted with antifading MOWIOL 4–88 (Calbiochem) with 2,5% Diazabicyclo [2.2.2] octane (DABCO) (Sigma) and observed with an inverted fluorescence microscope Eclipse TE2000-S (Nikon; Japan) using a Nikon 20/0.5 Plan Flour and 60/1.40 PlanApo oil immersion objectives. Images were taken with High-Definition Color Camera head DS-Fi1 (Nikon) controlled with Standalone Control Unit DS-L2 (Nikon). Images were merged, analyzed and edited for bright and contrast with ImageJ ver. 1.45 [31].

### RhoA Detection

RhoA activation was tested by the RhoA Activation Assay Kit (Cytoskeleton; Denver, CO) used according to the recommendations of the manufacturer. In brief, MA104 cells were grown on 6 well-plates to 80–90% confluency. Cells were infected with DxRRV (m.o.i. of 10), and tested at different times after infection (0.5, 1, 3, 5, 7 h.p.i.). At the indicated time, DxRRV and mock infected cells were washed with ice-cold PBS and lysed in ice-cold Lysis Buffer (50 mM Tris pH 7.5, 10 mM MgCl_2_, 0.5 NaCl and 2% Igepal), with the addition of a 1∶100 final dilution of a protease inhibitor cocktail (62 µg/mL leupeptin, 62 µg/mL pepstatin A, 14 mg/mL benzamidine and 12 mg/mL tosyl arginene methyl ester). Lysed cells were harvested with a cell scraper and clarified by centrifugation (10,000 rpm, 2 min, 4°C). All the samples were aliquoted and quick frozen by immersion in liquid nitrogen and stored at −70°C until used. Phosphorylated RhoA was pulled-down by incubation with rhotekin-RBD beads for 1 h at 4°C on a rotator, followed by 1 min centrifugation at 5000 x *g* at 4°C. Beads were washed with wash buffer (25 mM Tris pH 7.5, 30 mM MgCl_2_, 40 mM NaCl), and pelleted by centrifugation (5000 x *g,* 3 min, 4°C). Beads were finally resuspended with 2X Laemmli Sample Buffer, boiled for 2 min, spinned again and the supernatant analyzed by 12% SDS-PAGE and Western blotted with a anti-RhoA mAb antibody. Positive and negative control samples were obtained by treatment of MA104 cell clarified lysates with 20 mM GTPγS (a non-hydrolysable GTP analog) and 100 mM GDP stock, respectively.

### Treatment of DxRRV Virus Particles with Anti-VP5* Monoclonal Antibodies

DxRRV virus particles treated with 10 µg/mL porcine trypsin were incubated with a saturating concentration (5–10 µg/mL) of the non-neutralizing anti-VP5* HS2 monoclonal antibody (mab) for 1 h at 37°C (a kind gift of H.B. Greenberg, Stanford University, Palo Alto, CA). After the incubation, virus preparations were diluted with MEM to obtain a m.o.i of 10, and MA104 cell monolayers grown in 6 well-plates, inoculated for 1 h at 4°C on a rotary shaker. After the incubation, the inoculums was removed and cells were washed twice with PBS and incubated with new MEM medium at 37°C under a 5% CO_2_ atmosphere for different times after infection (0.5, 1, 3, 5, 7 h.p.i.). Activated RhoA detection and microfilament staining was carried out as described above.

### BAPTA-AM Treatment

MA104 cells infected with DxRRV were loaded with BAPTA-AM (1,2-bis(o-aminophenoxy)ethane-N,N,N’,N’-tetraacetic acid ester) (Molecular Probe, Invitrogen) to chelate intracellular Ca^2+^. Loading with BAPTA was performed as described previously [18] with few modifications. Briefly, MA104 cells were infected with DxRRV as described above and at 5 h.p.i. cultured media was removed and replaced with new fresh MEM medium containing 25 µM BAPTA-AM for 2 h at 37°C in a 5% CO_2_ atmosphere. Immunofluorescence assays were performed as described above. Higher concentrations of BAPTA-AM could not be used due to marked toxicity on the cells.

### siRNA Transfection

MA104 cells were treated with siRNA specific for rotavirus NSP4 gene as previously described [11]. Duplex siRNA^NSP4^ sequence 5′-AAACGUCAAAGUGUUCAUAUA-3′ (corresponding to nucleotides 219–239 of RRV gene 10) and control siRNA^lamin A/C^ were purchased from Dharmacon Research, Inc. (Dharmacon, Thermo Fisher Scientific Inc; Rockford, IL). MA104 cells were grown in 6 or 12 well-plates (with or without cover glass circle) and when the monolayers reached 70–80% confluency, the medium was removed and cells were washed twice with PBS. Transfection mixture consisted of 71.5 pmol of duplex siRNA (siRNA^NSP4^ or siRNA^lamin A/C^) and 0.25% Lipofectamine 2000 (Invitrogen) in OptiMEM (Gibco, Invitrogen). Transfection mixture was previously incubated for 30 min at room temperature before added to each well. MA104 cells were incubated with the transfection mixture for 4–5 h at 37°C in a 5% CO_2_ atmosphere. After this time, the transfection medium was replaced by fresh MEM supplemented with 10% FBS. Medium replacement, with fresh MEM was repeated at 15 h post transfection (h.p.t.). Transfected cells were infected with DxRRV (m.o.i. of 10) at 36 h.p.t. Immunofluorescence assays were performed as described above.

## Results

### Actin Microfilaments Organization is Altered in Rotavirus-Infected MA104 Cells

**Figure 1:**
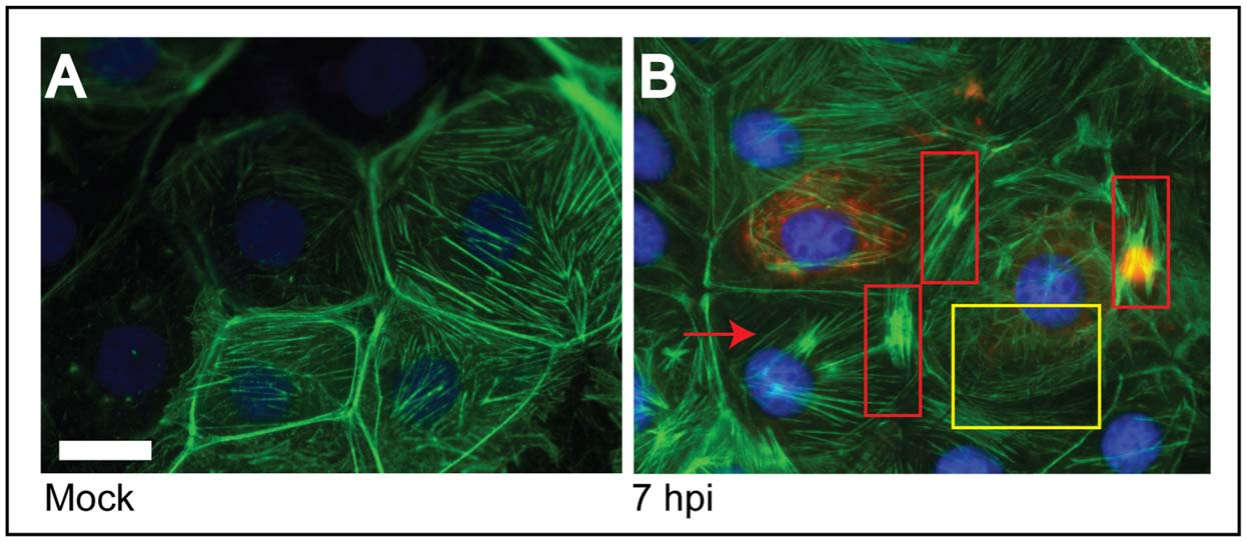
Changes in the actin cytoskeleton of MA104 cells infected with rotavirus. Cells were either mock infected (A) or infected (B) with the reassortant rotavirus strain DxRRV at an m.o.i. of 10; at 7 h.p.i. cells were fixed and processed for immunofluorescence. Actin was visualized using phalloidin-Alexa 488 (green) and nuclei were stained with DAPI (blue). Infected cells were detected using a anti-NSP4 (mAb) as primary antibody and an anti-mouse rhodamine-conjugated secondary antibody (red). Disorganized actin and stress fibers in infected cells are shown within yellow and red squares, respectively. Red arrows show actin stress fiber. Scale bar, 5 µm.

Since it is well established that rotavirus alters the [Ca^2+^]_cyto_ and that the cytoskeleton elements are Ca^2+^-dependent, [22], [25] we wanted to evaluate the state of the cytoskeleton proteins in rotavirus infected MA104 cells. Changes in actin microfilaments organization were studied by fluorescence microscopy using Alexa 488-labeled phalloidin toxin and DAPI for nuclei counter stain (Fig. 1). Mock-infected cells used as controls, showed a regular array of defined actin filaments present along the cells, evenly distributed in the cytoplasm, and also forming a well defined cortical ring under the plasma membrane (Fig. 1A). In contrast, by 7 h.p.i., DxRRV rotavirus-infected cells showed a marked disorganization of actin filaments, including a marked loss of actin at the cell periphery (Fig. 1B). In rotavirus infected cells defined actin filaments were not observed. Instead, a fibrillar or reticular actin filament pattern (Fig. 1B, yellow square), and aggregations of actin filaments that evoked actin stress fibers (Fig. 1B, red squares) were observed. The results are consistent with previous reports that indicate alterations in actin network in rotavirus-infected cells [24], [32], [33].

### Rotavirus Induces Changes in the Organization of Actin Filaments From a Very Early Stage of the Infection

**Figure 2:**
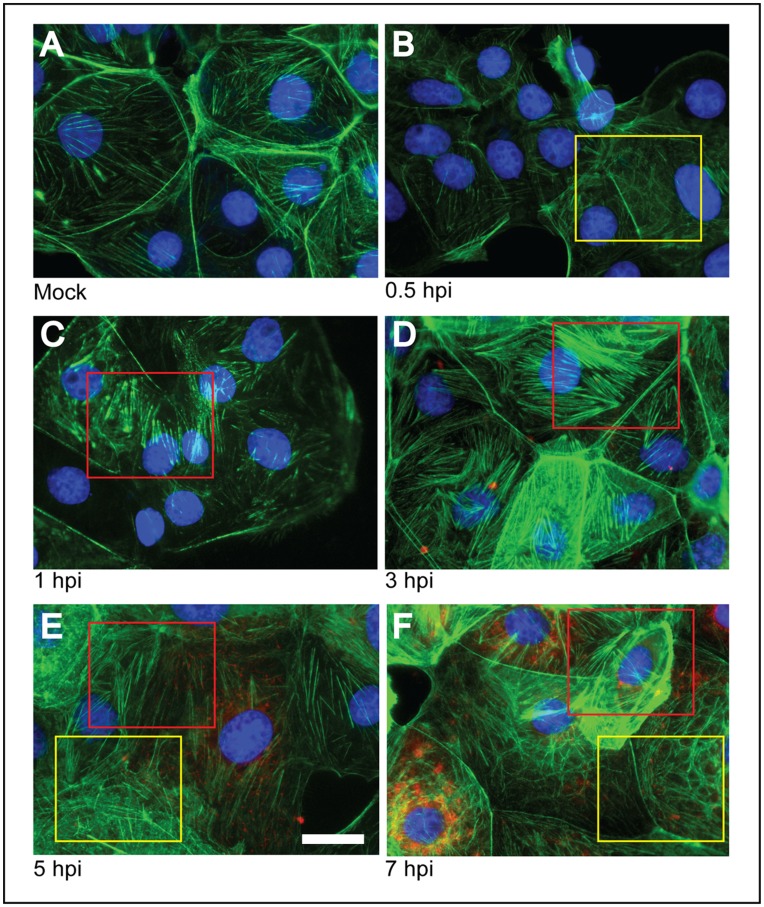
Time course of the changes in the actin cytoskeleton of MA104 cells infected with rotavirus. Cells were either mock infected (A) or infected (B–F) with the reassortant rotavirus strain DxRRV at an m.o.i. of 10. At the indicated h.p.i. cells were fixed and processed for immunofluorescence. Actin was visualized using phalloidin-Alexa 488 (green) and nuclei were stained with DAPI (blue). Infected cells were detected using a mAb anti-NSP4 as primary antibody and an anti-mouse rhodamine-conjugated secondary antibody (red). Disorganized actin and stress fibers in infected cells are shown within yellow and red squares, respectively. Note that actin disorganization is visible as early as 0.5 h.p.i and stress fibers after 1 h.p.i. and become clearly evident by 3 h., while NSP4 expression is detected after 5 h.p.i. Scale bar, 5 µm.

To monitor the changes observed in the distribution of actin filaments, and to determine at which stage of rotavirus infection these changes occur, fluorescence microscopy at different times of infection was performed (Fig. 2). Changes in the distribution of actin filaments were observed from very early stages of infection (Fig. 2B). Already at 0.5 h.p.i. actin filaments were observed reorganized into a fibrillar pattern (Fig. 2B, yellow squares). At 1 h.p.i. aggregations of actin and the appearance of actin structures compatible with stress fibers became evident (Fig. 2C, red square), and the disorganization observed at 0.5 h.p.i apparently disappears. At 3 h.p.i. the actin stress fiber-like structures were clearly visible (Fig. 2D). At 5 h.p.i, the expression of the NSP4 protein started to be detected (Fig. 2E, red stain). At this time of infection, actin reorganized into reticular arrays is observed along the actin stress fiber-like structures. Most dramatic changes were observed at 7 h.p.i. (Fig. 2F) when it seemed that the actin networks was totally collapsed.

### Rotavirus Infection Promotes Phosphorylation of RhoA

**Figure 3:**
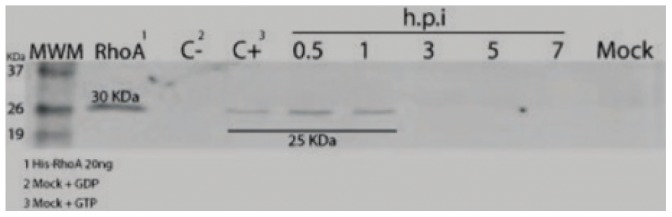
Activation of RhoA in MA104 cells infected with rotavirus. MA104 cells were infected with the reassortant rotavirus strain DxRRV at a m.o.i. of 10; at the indicated times, cells were lysed and analyzed by Western blotting to detect the presence of activated RhoA. MWM, molecular weight markers with protein weights indicated; RhoA, recombinant 6xHis-RhoA protein (20 ng) used as control; C− and C+, mock cells treated with GDP and GTPγS used as negative and positive controls respectively, for RhoA activation; h.p.i, hours post infection at which infected cells were processed; Mock, mock infected cell lysate used as negative control.

The formation of actin stress fibers is promoted by the activation of the small GTPase RhoA [34]. In order to determine if the actin array structures observed in DxRRV infected-cells are indeed actin stress fibers, the activation of RhoA was evaluated in rotavirus infected cells. With this aim, lysates obtained from a time course of rotavirus DxRRV infected-MA104 cells were used in a pulldown assay with rhotekin-RBD beads and phosphorylated RhoA detection was performed by western blot. The results indicate that rotavirus infection induces the activation of RhoA at early stages of infection (Fig. 3). Phosphorylated RhoA was detected at 0.5 and 1 h.p.i. However, no activated RhoA was detected at 3 h.p.i. and thereafter. No phosphorylation of RhoA was observed in mock-infected control cells, thus suggesting that rotavirus infection results in the activation of the RhoA protein that induces the subsequent formation of actin stress fibers [35].

### RhoA Activation and Stress Fiber Formation are Prevented in Cells Infected with Virion Antibody Complexes

**Figure 4:**
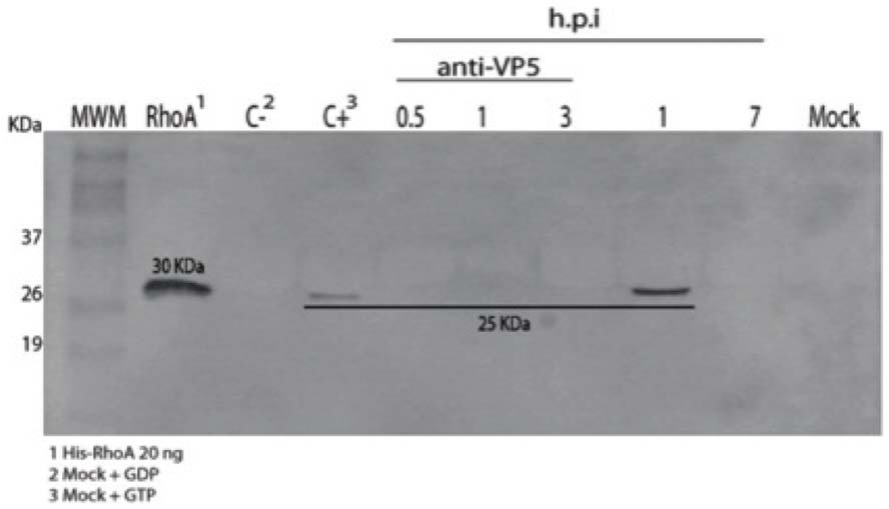
Prevention of RhoA activation in MA104 cells infected with rotavirus previously treated with anti-VP5* non-neutralizing mAbs. MA104 cells were infected with the reassortant rotavirus strain DxRRV previously treated (1 h at 37°C) or not with an excess of the anti-VP5* non-neutralizing mAb HS2; at the indicated times, cells were lysed and analyzed by Western blotting to detect the presence of activated RhoA. MWM, molecular weight markers with protein weights indicated; RhoA, recombinant 6xHis-RhoA protein (20 ng) used as control; C− and C+, mock cells treated with GDP and GTPγS used as negative and positive controls respectively, for RhoA activation; h.p.i, hours post infection at which infected cells were processed, lanes corresponding to the cell infected with the anti-VP5* mAb treated virus are indicated; Mock, mock infected cell lysate used as negative control.

**Figure 5:**
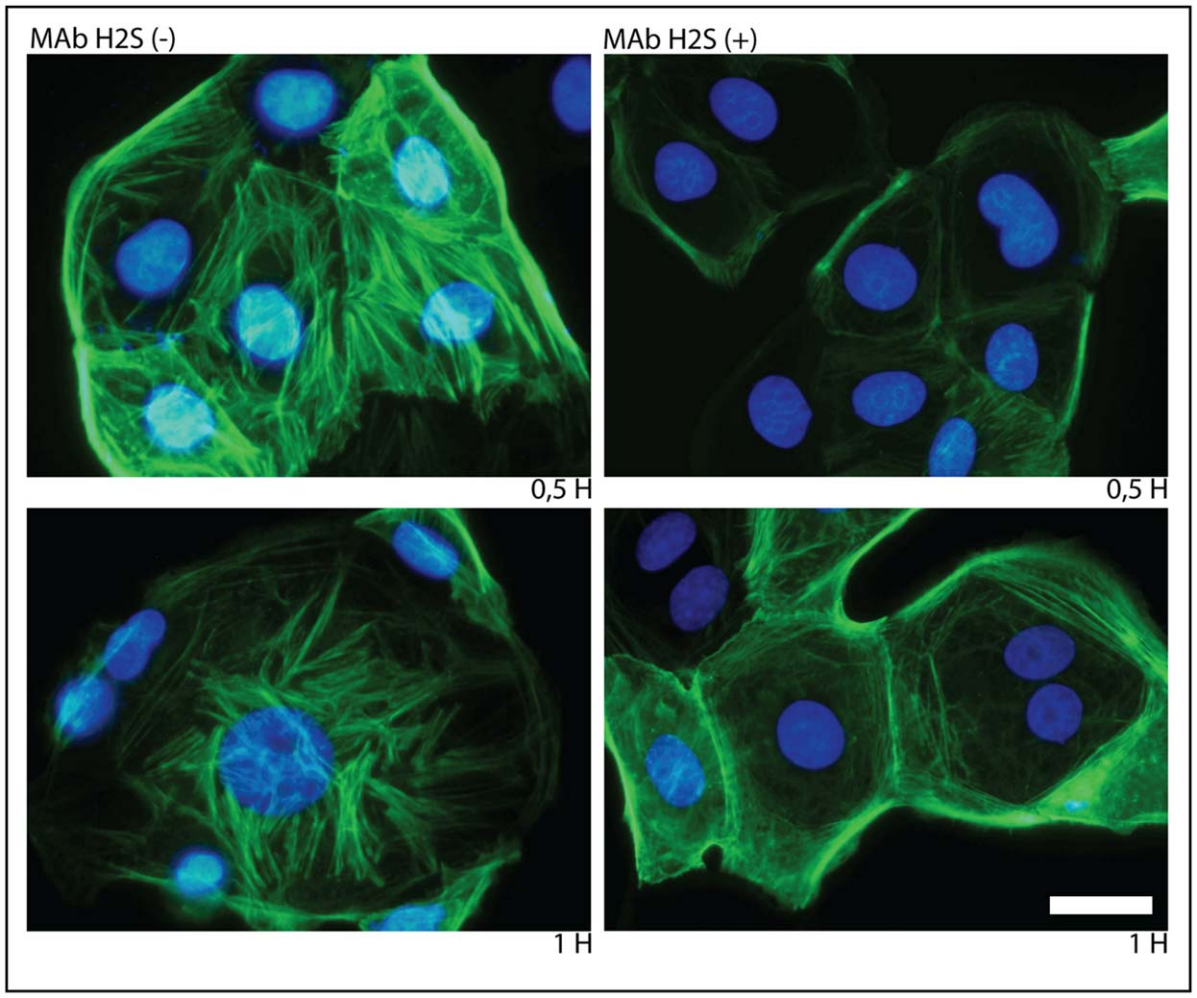
Prevention of stress fibers formation in MA104 cells infected with rotavirus previously treated with anti-VP5* non-neutralizing mAbs. MA104 cells were infected with the reassortant rotavirus strain DxRRV previously treated (HS2+) or not (HS2-) with an excess of the anti-VP5* non-neutralizing mAb HS2. At 0.5 and 1 h.p.i., cells were fixed and stained to visualize the actin cytoskeleton. Scale bar, 5 µm.

A large and diverse variety of cell surface agonists utilize Rho GTPases to modify cellular behavior. One of the ligand-activated receptors known to regulate RhoA activation are proteins of the integrin family. Several integrins have been reported to be part of the rotavirus receptor complex [36]. The rotavirus attachment protein VP5* contains recognition domains for the integrins α2β1 and α4β1 [37] whereas VP7 contains recognition domains for αxβ2 and αVβ3 [37], [38]. To investigate if RhoA activation is mediated by the interaction of the rotavirus virion with cell surface integrins, the phosphorylated state of RhoA was examined in cells infected with rotavirus virions previously reacted with H2S (mab), an anti VP5* non-neutralizing antibody. The results show that while RhoA activation was observed at 1 h.p.i. in cells infected with control virions, no activation was observed in cells infected with the antibody-virion complexes followed up to 3 h.p.i. (Fig. 4). Moreover, the formation of stress fibers or disassembly of the cortical actin were not observed in cells infected with virion-antibody complexes and fixed up to 1 h.p.i. (Fig. 5). These results taken together suggest that the activation of RhoA observed early after infection is promoted by the interaction between the virion and cell surface integrins.

### Treatment of Infected Cells with a Calcium Chelator or siRNA for NSP4 Prevents Actin Microfilaments Changes

**Figure 6:**
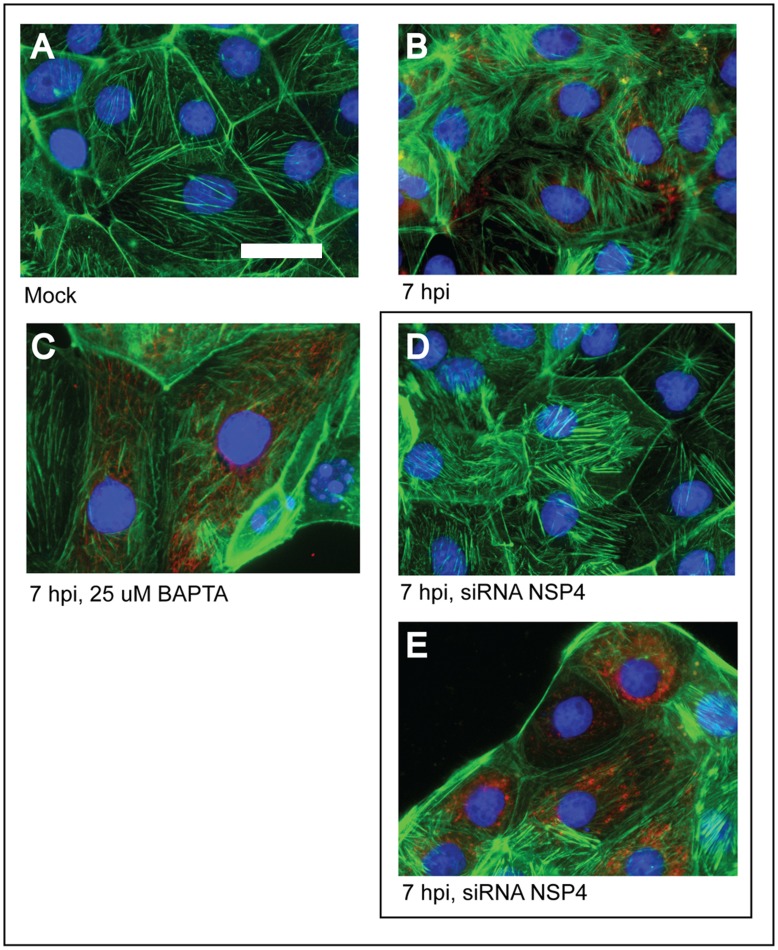
Effect of calcium chelation or silencing the expression of NSP4 on changes in the actin cytoskeleton of MA104 cells infected with rotavirus. Cells were either mock infected (A) or infected (B–E) with the reassortant rotavirus strain DxRRV at a m.o.i. of 10; at 7 h.p.i. cells were fixed and processed for immunofluorescence. Infected cells were either left untreated (B), treated with the indicated final BAPTA concentration 2 h before fixation (C), or transfected with siRNA^NSP4^ before infection (D and E). Infected cells transfected with siRNA^NSP4^ were stained either with antibody to NSP4 (D) or to VP6 as primary antibody (E). Note the presence of stress fibers, but not disorganized actin, in cell either treated with BAPTA or transfected with siRNA^NSP4^. Scale bar, 5 µm.

Since certain changes in the actin cytoskeleton are the results of changes in the [Ca^2+^]_cyto_, it was tested whether the observed microfilament changes would be reversed either by using an intracellular calcium chelator or by silencing the expression of NSP4, the viral protein mainly responsible for alteration of the cell calcium homeostasis. The results indicate that both intracellular BAPTA treatment as well as the suppression of NSP4 expression results in partial reversal of the disruption of the actin cytoskeleton (Fig. 6). However, stress fibers were observed in either BAPTA treated or NSP4 silenced cells. The 80% of rotavirus infected-cells treated with BAPTA, showed the actin stress fibers, and a similar proportion in those treated cells for silencing expression of NSP4. It should be noted that the mock cells showed less than 5% of stress fibers (Figure S1). These results indicate that the disruption of the actin cytoskeleton and the formation of stress fibers are triggered by different events and changes in the [Ca^2+^]_cyto_ are only responsible for the former, but are not related to stress fiber formation.

### Microtubules are Altered in Rotavirus-infected MA104 Cells

**Figure 7:**
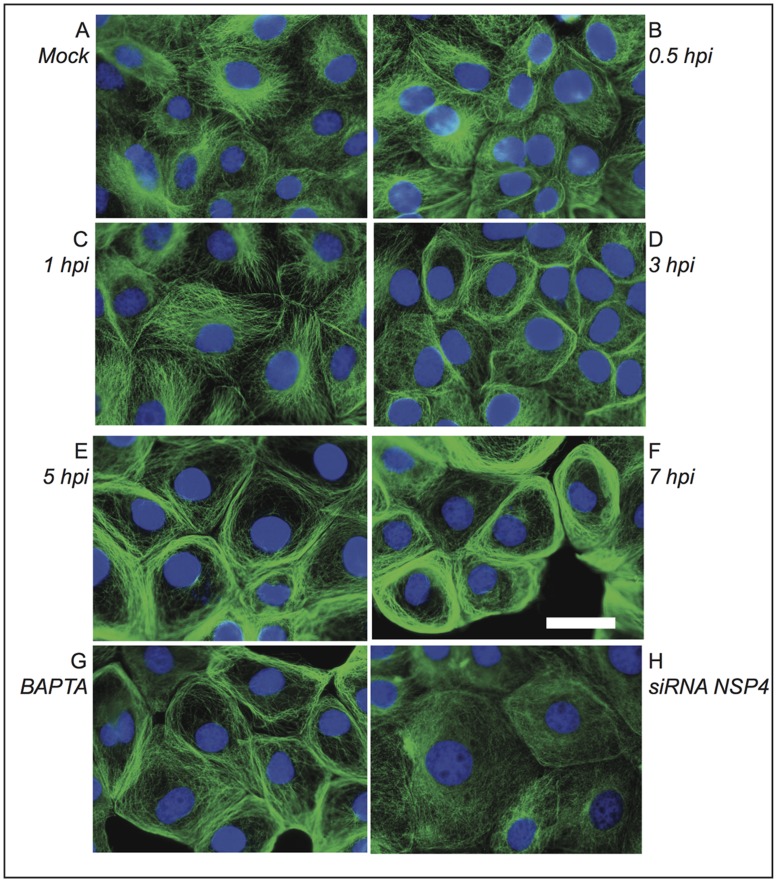
Changes in the tubulin cytoskeleton of MA104 cells infected with rotavirus. Cells were either mock infected (A) or infected (B–H) with the reassortant rotavirus strain DxRRV at an m.o.i. of 10. At the indicated h.p.i. (B–F) cells were fixed and processed for immunofluorescence. Tubulin was visualized using a mAb anti-α tubulin as primary antibody and an anti-mouse FITC-conjugated secondary antibody (green). Nuclei were stained with DAPI (blue). Note that tubulin disorganization is apparent after 3 h.p.i. In addition, infected cells were treated with 25 µM BAPTA 2 h before fixation (G), or transfected with siRNA^NSP4^ before infection (H) and fixed and processed for immunofluorescence at 7 h.p.i. Note that tubulin disorganization is partially or totally prevented in cell treated with BAPTA or transfected with siRNA^NSP4^, respectively. Scale bar, 5 µm.

To further explore the effect of the rotavirus infection on the cytoskeleton organization, experiments were also conducted to visualize the tubulin cytoskeleton of MA104 infected cells. Changes were observed in the organization of the tubulin cytoskeleton of MA104 cells when compared with control mock infected cells (Fig. 7). A marked displacement of the microtubular network toward the cell periphery started to be seen at 3 h.p.i. and was quite evident by 7 h.p.i. (Fig. 7A to 7E). The fibrillar appearance of the microtubules distributed throughout the cytoplasm was lost and replaced by filament bundles with increased fluorescence intensity at the cytoplasm periphery, and apparently close to the cell plasma membrane. Western blot experiments indicated that these changes occurred without marked changes in the tubulin concentration of the cell (data not shown). In order to evaluate if the observed changes in the tubulin cytoskeleton were related to changes in the cytoplasmic free Ca^2+^ concentration, infected cells were either loaded with the calcium chelator BAPTA or transfected with siRNAs to silence the expression of NSP4, and the structure of the tubulin cytoskeleton analyzed at 7 h.p.i. After BAPTA-AM treatment, partial reversion of the marginal distribution of microtubules was obtained, observing tubulin fibers filling the cytoplasm, although increased fluorescence intensity at the cell periphery was still detected (Fig. 7F). The reversal effect was more marked in cells where the expression of NSP4 was silenced (Fig. 7H). In these cells tubulin fibers were observed extended throughout the cytoplasm and no increase in fluorescence was observed at the cell periphery.

**Figure 8:**
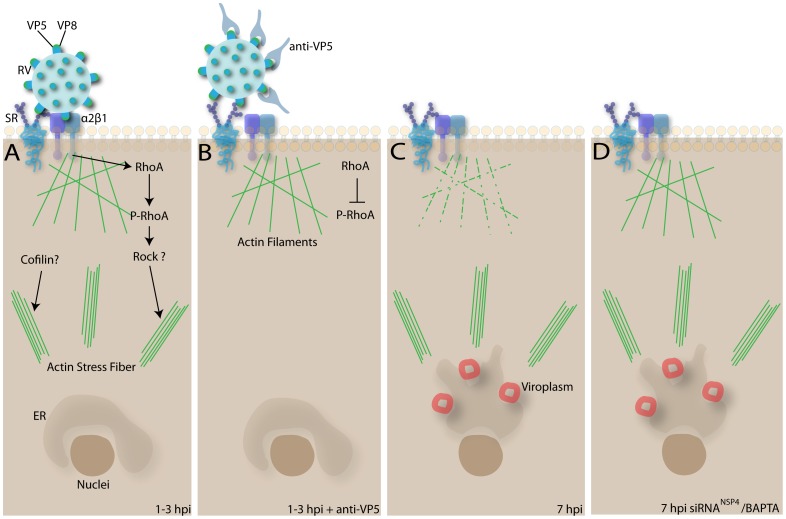
Summary of the actin perturbations in rotavirus infected cells. A: Early interaction of rotaviruses with α2β1 integrin trigger the phosphorilation of RhoA, which would lead the formation of actin stress fibers. The participation of Rock-1 and Cofilin in this process remains to be determined. The distribution of actin filaments is not affected at this time. B: The no interaction of VP5* with α2β1 integrin prevents the phosphorilation of RhoA and the appearance of actin stress fibers. C: At 7 h.p.i. actin filaments are disorganized and actin stress fibers are observed, although phosphorolated RhoA is no longer detected. D: Silencing of rotavirus NSP4 protein or treatment with the Ca^2+^ chelator BAPTA, results in reorganization of the actin filaments. However, the results suggest that neither the activity of NSP4 nor increased intracytoplasmatic Ca^2+^ levels have a role on the formation of actin stress fibers. RV: Rotavirus. SR: Sialic Receptor.

## Discussion

Previous reports have shown that rotavirus infection induces changes in the cell cytoskeleton. It has been shown that viral infection changes the dynamic morphology and structure of cytoskeletal components such as microtubules, keratins 18 and 8, vimentin (intermediate filaments) [28] and also the actin filaments. Actin filament modifications, which are sensitive to changes in calcium concentration, have been related to rotavirus proteins such as protein VP4. These changes have been observed in a variety of cell lines including MA104, Caco 2, Cos-7, HT29, CV-1 and BSC1. Moreover, rotavirus proteins VP4 [39], NSP2, NSP5 [40] and NSP4 [8] had been reported to interact directly with cytoskeletal proteins, specifically tubulin. In the studies reported here, the goal was to further evaluate the alterations of cell cytoskeleton, namely the microfilaments and microtubules, and to explore whether these alterations are related to the activity of the NSP4 protein, the enterotoxin responsible for the increase in cytoplasmic calcium concentration observed in cells infected with rotavirus. The results obtained confirm and extend previous reports indicating that rotavirus-infected MA104 cells present marked changes in the distribution of both the actin filaments and microtubules network. In addition, direct evidence that NSP4 expression and alterations in [Ca^2+^]_cyto_ are related to the observed changes in the cytoskeleton is presented. Furthermore, data presented show that Rho-A activation takes place early during rotavirus infection, inducing the formation of stress fibers and that such activation seems to be triggered by the interaction of infecting virions with cellular co-receptors.

Two kinds of changes in the actin filaments were found in infected MA104 cells; the formation of actin bundles in infected cells and a rearrangement of actin fibers into a fibrillar or reticular pattern. Time course experiments indicated that changes in the microfilaments started as early as 30 min after infection. Later on, between 1 and 3 h.p.i., the formation of actin bundles is observed, suggesting that the observed initial alterations of actin may be rearrangement of the actin network leading to the formation of actin bundles. However, the treatment of infected cells with BAPTA as well as the inhibition of NSP4 expression with siRNA to prevent an increase in intracellular calcium levels, showed a differential effect on both structures. In agreement with previous results [22], [25], [26], the rearrangements of actin into reticular patterns did not occurs when the increase of intracellular calcium levels was prevented by calcium buffering with the intracellular chelator. However, no effect was observed on the formation of the actin bundles, suggesting that this event is not associated with the alterations in the intracellular calcium homeostasis.

The actin bundles observed at the early stages of infection are reminiscent of actin stress fibers. Stress fibers are actin filaments that cross the cell and attach at focal adhesion plaques at the plasma membrane and to the network of intermediate filaments that surround the nucleus. Stress fibers have been linked to a variety of structural and physiological functions and their induction has been observed during infection with other RNA viral groups, including Dengue Virus [41] and Hepatitis C Virus [42]. The dynamics of the actin cytoskeleton, including the formation of actin stress fibers, is regulated by members of the RhoA family of small GTPases, namely Rac, Cdc42 and RhoA [34]. RhoA phosphorylation was detected in infected cells at 0.5 and 1 h.p.i., thus confirming that the actin bundles observed at early stages of infection are indeed actin stress fibers [43]. Since one of the integrins involved in the phosphorylation of RhoA is α2β1 [44], [45] [42], [43] [42], [43] [45], [46], which is part of the rotavirus receptor complex recognized by the VP5* protein [37] and no phosphorylation of RhoA was observed if virions were reacted with anti-VP5* antibodies, it is reasonable to presume that the activation of RhoA is due to virus-cell attachment. In agreement, Berkova et al. (2007) did not detect the phosphorylation of RhoA in cells expressing EGFP-NSP4 even though alterations in the actin network concomitant with alterations in intracellular calcium levels were observed [26]. On the other hand, integrins α1β1 and α2β1 have been reported to be the potential membrane receptors for the NSP4 enterotoxin [46], and apical addition of NSP4 perturbs actin distribution in polarized MDCK-1 cells [47]. Thus, it would be interesting to investigate if the binding of NSP4 to adjacent non-infected cells will result in RhoA stimulation in those uninfected cells. NSP4 is a viral enterotoxin [48]. Other microbial toxins able to induce diarrhea such as *E. coli* CNFs (cytoxic necrotizing factors) and *C. difficile* Toxin B have been associated with RhoA-induced activation as well as the formation of actin stress fibers [34], suggesting a role for these toxin induced alterations in the cytopathic effect and modifications of regulatory proteins in host cells.

Changes in the microtubule network in MA104 rotavirus infected cells very similar to the ones observed in this work, namely packing of the microtubules at the periphery of the cell cytoplasm, were reported recently by Martin et al. (2010) [49]. Based on the fact that changes in the tubulin network are observed in cells transfected with NSP2 alone, the authors concluded that NSP2 is the main factor for the microtubule reorganization observed in infected cells [49]. At variance, the results presented in this study indicate that changes in the intracellular calcium concentration induced by the expression of NSP4 are mainly responsible for the changes in the microtubule network observed in infected cells. However, it must be noted that the conclusions are not mutually exclusive, since neither the expression of NSP2 alone nor the silencing of NSP4 can fully restore the microtubule phenotype observed in non-infected cells. Thus, the dramatic changes observed in the microtubule network of rotavirus infected MA104 cells may be an additive effect of both viral proteins acting in concert. On the other hand, contrasting results have been reported regarding tubulin arrangements in rotavirus infected BCS-1 cells. While no major changes in the tubulin network were observed by Cabral-Romero and Padilla-Noriega (2006), changes very similar to the ones reported here were observed by Yang and McCrae (2011) after infection or expression of NSP4 [40], [50]. Moreover, and at variance with this study, Yang and McCrae (2011) report that changes in [Ca^2+^]_cyto_ are not necessary to affect the observed changes in the microtubules network. Thus, it is clear that further experiments are required to fully understand the dynamics of the microtubule network in rotavirus infected cells.

The pathogenesis of rotavirus infection has been extensively studied due to the great disease burden attributable to rotavirus infections. Many of these studies have examined the physiological changes related to the increased permeability of the plasma membrane to calcium and to the increase in intracellular calcium concentration observed during the course of rotavirus infection [51]. Results presented herein show that early virus events and later disturbances of intracellular calcium concentration during infection are at the base of the disorganization observed in the components of the cell cytoskeleton. A summary of the activity of rotavirus over actin filaments in infected cells is provided in Figure 8. The epithelial barrier function of the intestine relies on proper tissue architecture and in the integrity of the apical junctional complex [52], and both aspects are regulated by the cytoskeleton. For example, RhoA activation, with the concomitant disruption of cortical actin and stress fibers formation, as well as microtubule dynamics are intimately associated with cell barrier functions and control of the paracellular secretory pathway [53], [54]. Rotavirus infection of polarized cells is known to alter paracellular permeability and to alter the distribution of several tight junction proteins [55]. Thus, the alterations induced in the cellular cytoskeleton architecture, could contribute to the morphological changes observed in the cytopathic effect reported in rotavirus-infected cells, and therefore constitute an important component of the mechanism of rotavirus associated diarrhea.

## Supporting Information

Figure S1 **Distribution of actin in DxRRV-infected cells and DxRRV-infected cells treated with BAPTA and siRNA NSP4.** Cells were DxRRV-infected (A) and DxRRV-infected treated with BAPTA and siRNA NSP4 (F,K). At 7 h.p.i. DxRRV-infected cells and mock-infected cells (P), were fixed and processed for immunofluorescence. Actin filaments were stained with Alexa 586-phalloidin. Rotavirus VP6 protein was labeled with mAb 4B2D2 and detected with anti-mouse Alexa 488 (D, I, H, S). DAPI-stained nuclei are shown as a merge images for Alexa 586-phalloidin and VP6 (E, J, O, T). Details of actin phenotypes for each treatment are magnified (B, C, H, L, M, Q, R). Scale bar, 50 µm.
(TIF)
