# Supplementary figures and images for: Rotavirus Infection of Cells in Culture Induces Activation of RhoA and Changes in the Actin and Tubulin Cytoskeleton
Source: PLoS One. 2012 Oct 17;7(10):e47612. doi: 10.1371/journal.pone.0047612 (PMC3474729; doi:10.1371/journal.pone.0047612)

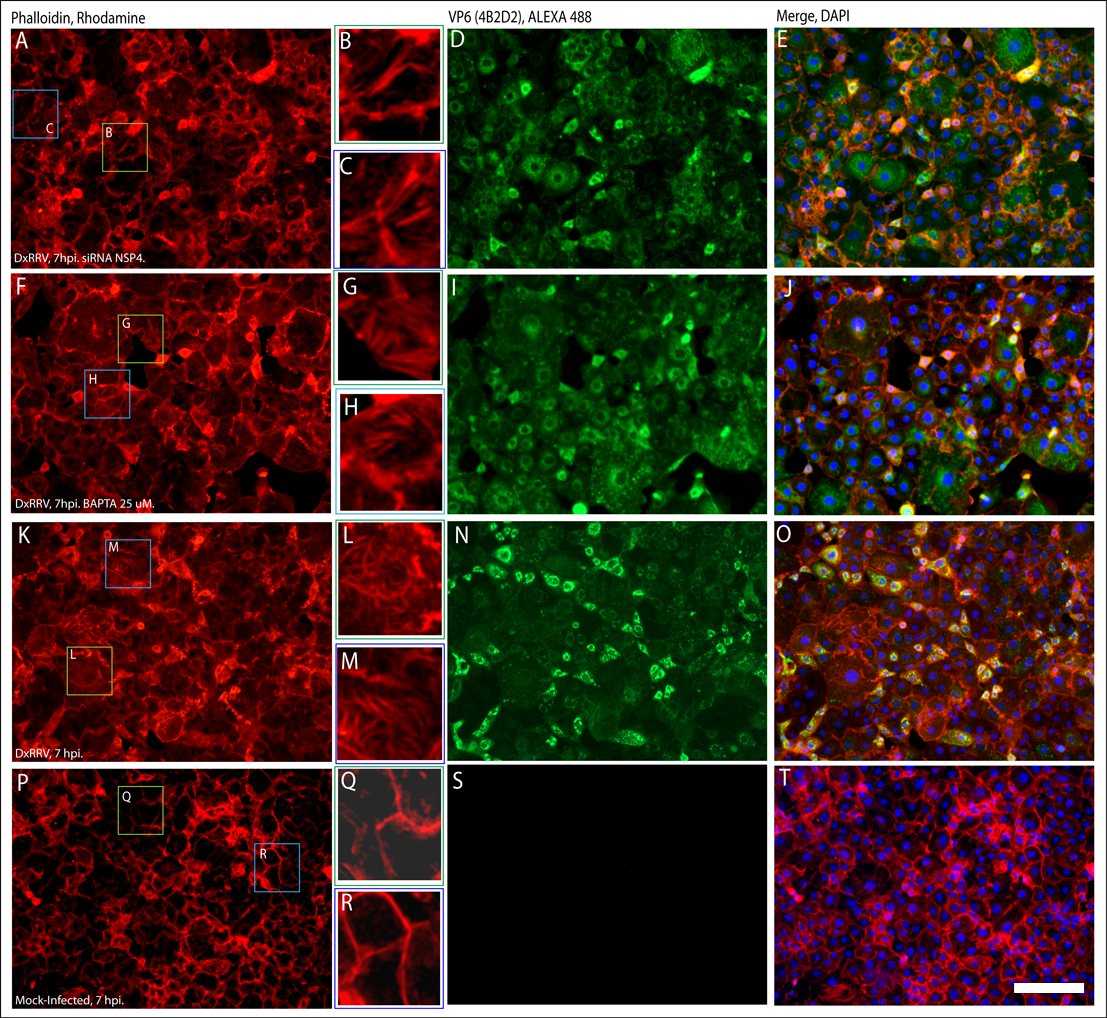

Supplement: Figure S1 — Distribution of actin in DxRRV-infected cells and DxRRV-infected cells treated with BAPTA and siRNA NSP4. Cells were DxRRV-infected (A) and DxRRV-infected treated with BAPTA and siRNA NSP4 (F,K). At 7 h.p.i. DxRRV-infected cells and mock-infected cells (P), were fixed and processed for immunofluorescence. Actin filaments were stained with Alexa 586-phalloidin. Rotavirus VP6 protein was labeled with mAb 4B2D2 and detected with anti-mouse Alexa 488 (D, I, H, S). DAPI-stained nuclei are shown as a merge images for Alexa 586-phalloidin and VP6 (E, J, O, T). Details of actin phenotypes for each treatment are magnified (B, C, H, L, M, Q, R). Scale bar, 50 µm. (TIF) [file pone.0047612.s001.tif]
